# The relationship between non-suicidal self-injury, identity conflict, and risky behavior among Druze adolescents

**DOI:** 10.3389/fpsyt.2022.938825

**Published:** 2022-11-09

**Authors:** Nermin Toukhy, Shir Ophir, Yelena Stukalin, Samer Halabi, Sami Hamdan

**Affiliations:** ^1^Department of Psychology, Bar Ilan University, Ramat Gan, Israel; ^2^School of Behavioral Sciences, Academic College of Tel-Aviv Yaffo (MTA), Tel Aviv-Yafo, Israel; ^3^Department of Psychological Medicine, Schneider Children's Medical Center, Petah Tikva, Israel

**Keywords:** Druze, adolescents, ethnic minority, identity conflict, non-suicidal self-injury

## Abstract

**Objectives:**

This study aimed to explore the prevalence of non-suicidal self-injury (NSSI) among Druze adolescents in Israel, an ethnic minority, and examine the influence of identity conflict, depression, and performance of risky behaviors on such adolescents' engagement in NSSI. This investigation is important because little is known about NSSI among adolescents from ethnic minorities.

**Methods:**

Overall, 290 Druze adolescents aged 16–18 (mean = 16.26, standard deviation = 0.9) years (63.9% female) participated in this study. They were recruited through snowball sampling from three Druze schools that agreed to participate in the study. All participants completed self-report measures for NSSI, depression, anxiety, engagement in risky behaviors, emotion regulation, sleep problems, and identity integration.

**Results:**

Almost 20% of the total sample engaged in NSSI. Those who engaged in NSSI reported more significant depression, anxiety, sleep problems, and engagement in risky behaviors when compared with those who did not engage in NSSI. Moreover, those who engaged in NSSI reported experiencing a higher level of identity conflict. Further analysis revealed an indirect effect of identity conflict on NSSI through engagement in risky behaviors.

**Conclusions:**

This study's findings clarify the prevalence of NSSI among Druze adolescents, as well as contributing factors, and also highlights the importance of developing interventions that specifically target this unique ethnic group.

## Introduction

Non-Suicidal Self-Injury (NSSI) refers to one deliberately inflicting damage, pain, or both on their own bodily tissue without suicidal intention (1). NSSI is mostly associated with cutting oneself; however, there are many other methods of deliberately causing harm to one's own body, such as carving, scratching, burning, or ripping of skin or hair, swallowing toxins, bruising, and breaking bones (2). When self-inflicted, these methods are considered forms of self-injury.

Many studies have found a significant linkage between NSSI and suicidal behavior and, accordingly, that repetitive NSSI increases risk of suicide (3). Moreover, the relationship between NSSI and attempted suicide has been found to be stronger than that between depression and attempted suicide (4). Other studies, however, have argued that NSSI is an independent pathology, and there have been consistent proposals to include NSSI as a disorder in the Diagnostic and Statistical Manual of Mental Disorders-5 (5).

The present study examines NSSI among Druze adolescents in Israel, a unique ethnic minority that is under-represented in current research on NSSI. Among Western populations of adolescents and college students, the lifetime prevalence of NSSI ranges from 13 to 35% (6) while in Israel, approximately 20% of adolescents report engaging in NSSI (7, 8).

Most existing investigations of NSSI have been conducted among Western cultures. Little research has been conducted among ethnic minorities and non-Western cultures. A recent review (9) that explored existing results concerning NSSI among ethnic minorities in Western countries (mainly African-Americans in the US) revealed that there are mixed findings regarding whether NSSI is more prevalent among ethnic minorities when compared to majorities. Some studies have found lower prevalence rates among ethnic minorities (38.7% among African Americans compared to 55.5% among Americans of European descent) (10), while others have found either no differences or higher prevalence rates [43% among African Americans vs. 37% among Americans of European descent (11)]. Most relevant to the current study, however, are findings that indicate that, among ethnic minorities, ethnic identity may act as a protective factor against NSSI (9).

Identity formation is an important developmental stage of adolescence (12), meaning it is important to examine the relationship between identity conflict and NSSI. Claes et al. (13) found a positive correlation between NSSI and identity confusion and depression, which suggests that NSSI is a maladaptive coping strategy for the challenges associated with developing a mature identity, and that identity confusion and depression are associated with a greater likelihood of engaging in NSSI. Similarly, Breen et al. (14) found that individuals who experience identity-development issues, which are associated with negative self-appraisal and painful emotions, may engage in NSSI in an attempt to manage these effects.

In accordance with the above findings, in the present study we aim to explore the prevalence of NSSI among Israeli Druze, a distinct ethnic minority in Israel, and determine whether ethnic identity conflict constitutes a unique risk factor for NSSI.

Israel's ethnic mosaic is complex, comprising a Jewish majority and an Arab minority, with the latter group including Christians, Muslims, and Druze. However, for both political and historical reasons, since the establishment of the state of Israel the Druze population in Israel has been considered a minority within a minority, separate from Israel's Arab population (15). Unlike other Arab adolescents, Druze adolescents join the Israeli Defense Forces (IDF) on a compulsory basis (16). As such, the Druze identity comprises three factors: Druze, which represents their primordial identity and usually relates to religion; Arab, which includes their use of the Arabic language and the Arab community's attitude toward Druze people; and Israeli, which involves their civilian status and duties, as well as the attitude of the state and the Jewish population toward Druze people (15).

Indeed, one of the major factors that shape the Druze identity in Israel is their obligatory service in the IDF. On the one hand, as a civic duty, army service is an integral part of their Israeli identity. On the other hand, it challenges the Druze's Arab identity, given that parts of the Arab society consider service in the IDF to be an act of treason (15).

Considering that the Druze element is the most fundamental in the Druze identity, we assume that the primary identity gap for the Druze people is between the Israeli and Arab elements. Moreover, given that army service begins at the age of 18, we also assume that this conflict begins to manifest prior to or during adolescence. Overall, we anticipate that, among Druze adolescents, the frequency of NSSI is greater among those who have a high identity conflict when compared to those with a low identity conflict.

Examinations of NSSI in ethnic minorities should also consider other clinical factors that have been found to be the most robust risk factors associated with NSSI. Previous studies have highlighted several potential clinical risk factors, including depression (17, 18), anxiety (17), emotion dysregulation (19), engagement in risky behaviors (7), and sleep problems (20). The latter clinical risk factors are known to be prominent among adolescents in minority groups. Previous research indicate that those risk factors are heavily influenced by minority stress, such as discrimination, socioeconomic status, daily hardships, internalization of negative events and acculturation stress (21). Aggression, impulsivity, engagement in risk behaviors and emotion dysregulation are thought to have a greater influence on self-injury among minority compared to majority adolescents (21). Sleep problems and depression, that are both well-known risk factors for self-injury, are prevalent among ethnic minority adolescents as well (22, 23). Thus, in the present study we focus on these as key variables and examine them among Druze adolescents.

Sleep problems have been found to be strongly associated with NSSI among adolescents (24). More specifically, a recent systematic review (24) revealed that sleep problems such as short sleep duration, sleep disturbances, and poor sleep quality are associated with NSSI, while emotional dysregulation and depression appear to mediate this relationship.

Emotion-regulation can be defined as an individual's attempt to moderate his/her emotions in real-time, how he/she experiences them, and how he/she expresses them. In particular, of the many attempts to explain the mechanism underlying NSSI, the emotion-regulation model is the most empirically supported (25). According to this model, NSSI represents a strategy for alleviating acute negative effects, and studies based on this model have indicated that while individuals experience high states of negative emotion before NSSI, this emotional state decreases during and after the self-injury act (26). It has also been found that those who engage in self-injury, when compared to individuals who do not engage in NSSI, experience negative emotions more frequently in their daily lives (6), and that NSSI is used to manage stress originating from intrapersonal and interpersonal difficulties, including low self-esteem and low self-efficacy (27).

Notably, various studies have demonstrated an association between emotional dysregulation and engagement in prototypical health risk behaviors such as alcohol consumption, tobacco and alcohol use, unsafe sex and dangerous driving. For instance, emotionally dysregulated individuals have been found to be more likely to engage in risk-taking behaviors when compared with controls, and studies also suggest that the desire to reduce negative emotions leads to engagement in risky behaviors (28). Further, increased impulsivity is related to increases in engagement in a variety of prototypical risky behaviors (29, 30). A notable finding in the context of the present study is that adolescents who engage in NSSI report greater involvement in risky behaviors than those who do not engage in NSSI (7). In this study, we aim to examine specific types of health risk behaviors (e.g., drink-driving, practicing unsafe sex) and to examine the relationship between engagement in those risky behaviors and NSSI.

The purpose of this study is to explore the prevalence of NSSI and risk factors for NSSI behaviors (i.e., symptoms of depression, anxiety, emotion dysregulation, engagement in risky behaviors, and sleep problems) among the adolescent Druze population in Israel. In addition, this study aims to examine the association between identity conflict and NSSI, and the influence of the abovementioned risk factors on this association. We hypothesize that (1) anxiety, depressive symptoms, sleep problems, identity conflict, emotional regulation, and involvement in risky behaviors are more frequent among those who engage in NSSI when compared with those who do not engage in NSSI; and (2) identity conflict is mediated by the relationship between depression and NSSI, in the sense that in the presence of depression and a high level of identity conflict, participants are more prone to engage in NSSI.

## Materials and methods

### Participants and procedures

**Figure 1:**
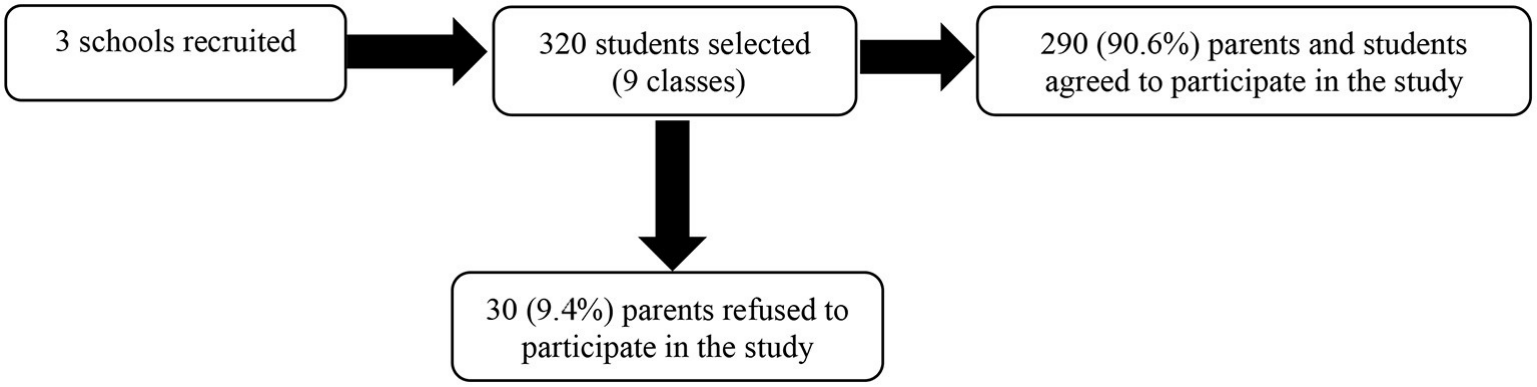
Sampling procedure flow chart.

Overall, 290 Druze adolescents aged 16–18 years participated voluntarily and anonymously in this study. The average age among the respondent sample was 16.28 (SD = 0.9) years. The participants were recruited from three Druze schools located in Druze villages. According to the Israeli Central Bureau of Statistics, 99.95% of the residents of these areas are Druze. Three classes were selected in every school, and a consent form was sent to 320 parents (Figure 1). The researchers informed students about an anonymous study exploring non-suicidal self-injury and other risk factors, which will take place in a particular day, instead of the class scheduled. Researchers sent a handout to parents of students, including information regarding the aims of the study. Parents were able to update the school administration or the researchers if they do not wish their children to participate in the study, and students whose parents did not agree they will participate were excluded. In total 9.4% of the parents refused to participate in the study (Figure 1). We could not obtain demographic or academic information regarding those who refused. After obtaining the appropriate informed consent materials from the school and students' parents, participants were provided, through a referral sheet, with information regarding mental health services, emergency hotlines, and local mental-health center contacts to allow them to discuss their feelings with support services, if they so wished. This study was approved by the Ethics Committee of the Ministry of Education of Israel and the institutional review board of the Academic College of Tel-Aviv-Yaffo and complied with APA ethical standards.

The primary demographic characteristics of the participant sample are listed in Table 1.

**Table 1:** Demographic and clinical characteristics of the sample.

	**N**	**%**
Gender (female)	129	63.9
Cohabitation status (Living with both parents)	197	94.3
Religiosity (religious)	135	64.3
Mother's education level (Academic education- above high school)	65	31.9
Father's education level (Academic education- above high school)	46	22.5
Engagement in NSSI^a^	41	19.7
Suicidal ideation	37	17.7
Sleep problems	122	58.9
Identity conflict	74	50
Moderate–severe symptoms of Depression	43	20.6
Moderate–severe symptoms of Anxiety	56	26.8

a NSSI, Non Suicide Self Injury.

### Measures

All questionnaires were presented to participants in Arabic, the participants' native language. The questionnaires collected clinical and demographic information.

#### Deliberate self-harm inventory-youth version

To measure the participants' engagement in self-harm, we included in the questionnaire a shortened and modified version of the Deliberate Self-Harm Inventory (31): the Deliberate Self-Harm Inventory-Youth Version ([SHI-Y; adapted for adolescents by Lundh et al. (32)]. In the DSHI-Y, respondents are asked whether they have deliberately engaged in one or several of nine types of self-harming behaviors during the past six months. Respondents report the number of times they engaged in these behaviors using a seven-point Likert scale ranging from 0 (“never”) to 6 (“more than five times”). The total score for the DSHI-Y (ranging from 0 to 54) is calculated by summing the scores for each behavior. In our research, based on Gratz (31) recommendation, we calculated a dichotomous binary variable, with “1” indicating having engaged in NSSI more than twice, and “0” otherwise. Regarding internal consistency for the DSHI-Y, for this study the Cronbach's α value was 0.9.

#### Athens insomnia scale

To measure the participants' sleep, we used the Athens Insomnia Scale [AIS; (33)]. This scale comprises eight items, of which the first five measure the quality and amount of sleep the respondent obtains each night, and the final three measure the effect of sleep on the respondent during the day. Each question is rated from 0 (“no problem”) to 3 (“very serious problem”). The total score is calculated by summing the scores for all questions and, thus, ranges from 0 to 24. Scores that are higher than or equal to six indicate the presence of insomnia; this cutoff point correctly distinguishes between individuals with insomnia and controls in 90% of cases (34). For this study, Cronbach's α for the AIS was 0.74.

#### Generalized anxiety disorder-7

To measure anxiety among the participants, we used the Generalized Anxiety Disorder-7 [GAD-7; (35)]. This seven-item inventory assesses the frequency by which respondents experience the core symptoms of generalized anxiety disorder. Respondents are asked how often they have experienced anxiety symptoms over the previous 2 weeks (e.g., “I felt so restless, I had trouble sitting without moving”). All answers are provided using a four-point Likert scale ranging from 0 (“not at all”) to 3 (“nearly every day”), with the total score ranging from 0 to 28. In our study, we used anxiety as both a dichotomous variable and a continuous variable. The dichotomous variable was defined using a cutoff point of 10; the continuous variable was defined as the sum of the participants' answers. For this study, the Cronbach's α for the GAD-7 was 0.90.

#### Patient health questionnaire modified for adolescents

To measure depression, we used the Patient Health Questionnaire modified for Adolescents [PHQ-A; (36)]. This is a depression inventory modified for adolescents. It comprises nine questions, each regarding a different aspect of depression, and enquires into how often the respondent has experienced each aspect of depression over the previous 2 weeks. Scores are given using a Likert scale ranging from 0 (“not at all”) to 3 (“almost every day”). In the present study, we analyzed the depression variable as both a dichotomous variable and a continuous variable. The dichotomous depression variable was defined using a cutoff point of 10; accordingly, participants who scored 9 or less were defined as not having experienced depression in the previous 2 weeks, while those who scored 10 or higher were defined as having experienced depression in the previous 2 weeks. Meanwhile, the continuous variable was calculated as the sum of the participants' responses for all items. Suicidal ideation was calculated using the final question on the PHQ-A, which asks whether the respondent has, within the previous 2 weeks, had “Thoughts that you would be better off dead or of hurting yourself in some way.” A participant was defined as having suicidal ideation if they responded “1” or higher to this question; those who responded “0” were defined as not having suicidal ideation. For the present study, the Cronbach's α for this inventory was 0.87.

#### Emotion-regulation questionnaire

To measure the participants' emotion regulation, we used the Emotion-Regulation Questionnaire [ERQ; (37)]. The ERQ comprises 10 items, all of which are answered using a Likert scale ranging from 1 (“strongly disagree”) to 7 (“strongly agree”). This questionnaire contains two major subscales: cognitive reappraisal and expressive suppression. The higher a participant's score, the greater his/her use of an emotion-regulation strategy. The measurement of cognitive reappraisal concerns whether the respondent changes the way he/she thinks about emotion-eliciting events (37). For this study, the Cronbach's α for the reappraisal subscale was 0.65. The measurement of expressive suppression concerns whether the individual changes how they express their emotions by suppressing their emotion-expressive behavior (37). For this study, the Cronbach's α for the suppression subscale was 0.66.

#### Bicultural identity integration scale

To measure identity conflict, we used the Bicultural Identity Integration Scale [BIIS-2; (38)]. The items on this scale are scored using a five-point Likert scale ranging from 1 (“strongly disagree”) to 5 (“absolutely agree”). The BIIS-2 comprises 15 questions concerning various aspects of identity; the items are designed to evaluate whether the respondent experiences a conflict between identity factors. We modified the names of the identities mentioned in the items to suit the context of the Druze participant, sample for example: “I feel trapped between my Druze identity and my Arab identity.” Respondents' scores for this questionnaire are obtained by summing the scores for all items. Lower scores indicate a more complete identity, while the maximum score of 75 indicates severe identity conflict. The total score for the BIIS-2 ranges from 15 to 75 and, in the current study, we used the median score for this range (39) as the cutoff point for determining the presence of identity conflict; thus, an individual whose score was equal to or higher than 45 was defined as having an identity conflict. To implement this approach, we created a dichotomous variable for the absence/existence of identity conflict. For this study, the internal consistency for this inventory, measured using Cronbach's α, was 0.80.

#### Modified risk involvement and perception scale

The Modified Risk Involvement and Perception Scale [M-RIPS; (40), with changes made by Ben-Zur and Reshef-Kfir (41)] is a 31-item scale that assesses respondents' frequency of involvement in risky behavior. Responses are given using a five-point Likert-type scale ranging from 0 (“not at all”) to 4 (“every day”). Each item concerns either a low-risk behavior (e.g., riding a motorcycle, taking money from parents without permission) or a high-risk behavior (e.g., drink-driving, practicing unsafe sex) displayed by adolescents. In this study, we used total scores for the items as a variable indicating involvement in risky behaviors, with higher scores reflecting more frequent engagement in risky behavior. This Cronbach's α for this questionnaire was 0.97.

#### Demographic information

The demographic information collected from the participants included age, class, parents' education, parents' birthplace, number of siblings, and religiosity level.

### Statistical analysis

Data analysis was conducted using IBM SPSS Statistics, version 25 (IBM Corp., Armonk, N.Y., USA). Participants who engaged in NSSI were compared with those who did not in terms of demographic and clinical characteristics using either chi-square tests or *t*-tests (depending on which was appropriate). The alpha level was set to 0.05. Regression diagnostic tests were used to assess the multicollinearity between predictors. Multiple logistic regression analysis was performed to test the predictive utility of factors that significantly correlated with engagement in NSSI. Goodness-of-fit statistics were used to compare and select the most parsimonious models. Bonferroni correction was administered, as the comparison included multiple tests. Based on the variables found in the final model in the regression analysis, we created mediation hypotheses. Then, to test our mediation hypotheses we used the PROCESS macro 3.3 for SPSS developed by Hayes [(42), 3^rd^ edition].

## Results

### Preliminary analysis

Clinically, 19.7% of the sample reported having engaged in NSSI on three or more occasions. Most of the respondents reported clinical sleep problems (58.9%, *n* = 122), 20.6% of the sample experienced moderate–severe symptoms of depression (*n* = 43), and 26.8% experienced moderate–severe symptoms of anxiety (*n* = 56). The sample's clinical characteristics are listed in Table 1.

To predict individuals with an increased risk of engaging in NSSI, we performed a *t*-test and chi-square test (for continuous and dichotomous variables, respectively) between the questionnaire results for the group who reported engaging in NSSI and the group that did not engage in NSSI. Compared to the non-NSSI group, the NSSI group contained a larger proportion of boys [50% versus 32.9%; χ^2^(*df* = 1) = 3.88, *p* = 0.49], indicating that in this sample boys were more prone to NSSI (see Table 2).

**Table 2:** Demographic characteristics in terms of engagement in non-suicidal self-injury.

	**Engaged in NSSI *(n = 41)***	**Did not engage in NSSI *(n = 167)***				
			**Test**	***df***	**P**	**Effect size**
Age (M ± SD)	16.07 ± 0.9	16.33 ± 0.9	t = −1.66	205	0.098	0.289
Gender (male) % (N)	50% (19)	32.9% (53)	χ^2^ = 3.88	1	0.049	−0.140
Religiosity (religious) % (N)	13.5% (28)	51.2% (106)	χ^2^ = 0.602	1	0.438	0.070
Living with both parents	17.5% (36)	76.7% (158)	χ^2^ = 1.57	1	0.209	0.087
Mother's education level (academic level) % (N)	24.3% (9)	33% (55)	χ^2^ = 1.13	2	0.567	0.075
Father's education level (academic level) % (N)	10.8% (4)	25.6% (42)	χ^2^ = 3.98	2	0.136	0.141
Sleep problems % (N)	78% (32)	52.6% (88)	χ^2^ = 8.03	1	< 0.01	0.198
Identity conflict % (N)	48.7% )20)	31.1% (52)	χ^2^ = 5.59	1	< 0.05	0.196
Emotion-Regulation suppression (M ± SD)	16.65 ± 5.2	15.77 ± 6.13	t = 0.82	184	> 0.1	0.155
Emotion-Regulation reappraisal (M ± SD)	22.4 ± 7.07	26.71 ± 9.85	t = −2.6	196	< 0.05	0.503
Engagement in risky behaviors (M ± SD)	20.24 ± 20.77	7.62 ± 14.5	t = 4.5	206	< 0.001	0.705
Depression (M ± SD)	11.29 ± 7.25	4.6 ± 4.18	t = 7.76	205	< 0.001	1.131
Anxiety (M ± SD)	10.82 ± 5.77	5.23 ± 4.46	t = 6.75	205	< 0.001	1.084

M ± SD, mean ± standard deviation; NSSI, Non-suicidal self-injury.

In terms of clinical characteristics, adolescents in the NSSI group, when compared to the adolescents in the non-NSSI group, showed a higher degree of identity conflict [48.7% (*n* = 20) vs. 31.1% (*n* = 52); χ^2^(*df* = 1) = 5.59, *p* < 0.05] and sleep problems [78% (*n* = 32) vs. 52.6% (*n* = 88); χ^2^(*df* = 1) = 8.03, *p* < 0.01], respectively. In comparison with the non-NSSI group, the members of the NSSI group showed severe symptoms of anxiety [t_205_ = 6.75, *p* < 0.01] and depression [t_205_ = 7.76, *p* < 0.01], and also showed higher engagement in risky behaviors [t_206_ = 4.5, *p* < 0.01]. Furthermore, the NSSI group presented a lower level of emotion-regulation reappraisal when compared to the non-NSSI group [t_196_ = −2.6, *p* < 0.05] (see Table 2).

Examination of the correlations between the risk factors mentioned above and NSSI showed that NSSI is significantly correlated with identity conflict (r = 0.17, *p* < 0.05), sleep problems (r = 0.40, *p* < 0.01), anxiety (r = 0.42, *p* < 0.01), depression (r = 0.49, *p* < 0.01), and engagement in risky behaviors (r = 0.48, *p* < 0.01). A significant negative correlation was also found between NSSI and emotion-regulation reappraisal (r = −0.11, *p* < 0.05). In addition, a significant partial correlation was found between NSSI and emotion-regulation suppression when controlling for gender (*r* = 0.16, *p* < 0.05). Full correlations as well as partial correlations (controlling for gender) are presented in Table 3. This latter finding confirms our second hypothesized correlation, that there is a negative association between emotion-regulation reappraisal and NSSI.

**Table 3:** Pearson full and partial^a^ correlations between variables.

		**1**	**2**	**3**	**4**	**5**	**6**	**7**	**8**
1	NSSI	1							
2	Emotion-Regulation Suppression	0.05 (0.16^*^)	1						
3	Emotion-regulation reappraisal	−0.11^*^ (0.06)	0.54^**^ (0.53^**^)	1					
4	Risky behavior	0.45^**^ (0.32^**^)	−0.23^**^ (−0.14)	−0.24^**^ (−0.13)	1				
5	Depression	0.49^**^ (0.53^**^)	0.101 (0.22^*^)	−0.06 (0.08)	0.24^**^ (0.20^*^)	1			
6	Anxiety	0.42^**^ (0.47^**^)	0.144^*^ (0.24^**^)	−0.08 (0.04)	0.18^**^ (0.18*)	0.72^**^ (0.76^**^)	1		
7	Sleep	0.40^**^ (0.51^**^)	0.09 (0.20^*^)	−0.08 (0.031)	0.22^**^ (0.24^**^)	0.49^**^ (0.52^**^)	0.53^**^ (0.60^**^)	1	
8	Identity conflict	0.17^*^ (0.07)	−0.123 (−0.07)	−0.141 (-0.10)	0.246^**^ (0.06)	0.047 (−0.05)	0.084 (−0.05)	0.051 (0.03)	1

* *p* < 0.05,

** *p* < 0.01.

a Partial correlations between variables when controlling for gender (presented in brackets). NSSI, non-suicidal self-injury.

Multiple logistic regression analysis was performed to test the predictive utility of the different variables in regard to NSSI. When controlling for the abovementioned demographic and clinical correlates that had been found to be significantly associated with NSSI, the most parsimonious model set was consequently found to include identity conflict (odds ratio [OR] = 3.23, *p* = 0.03, *ROC* = 0.596), depression (OR = 1.21, *p* < 0.01, *ROC* = *0.802*), and engagement in risky behaviors (OR = 1.03, *p* = 0.01, *ROC* = *0.757*). For the final step of the model, χ^2^ = 34.13, *p* < 0.001 (Table 4).

**Table 4:** Logistic regression results for the relationships among identity conflict, depression, and engagement in risky behaviors in regard to non-suicidal self-injury.

	**OR**	**SE**	**Wald**	**P**	**95% CI**	**ROC**	**χ2**	**R^2^**	***p***
Final model							34.13	0.36	< 0.0001
Identity conflict	3.23	0.56	4.32	0.03	1.07–9.75	0.596			
Depression	1.21	0.04	17.99	< 0.01	1.11–1.32	0.802			
Engagement in risky behaviors	1.03	0.01	5.93	0.01	1.01–1.05	0.757			

CI, confidence interval; OR, odds ratio; SE, standard error.

**Figure 2:**
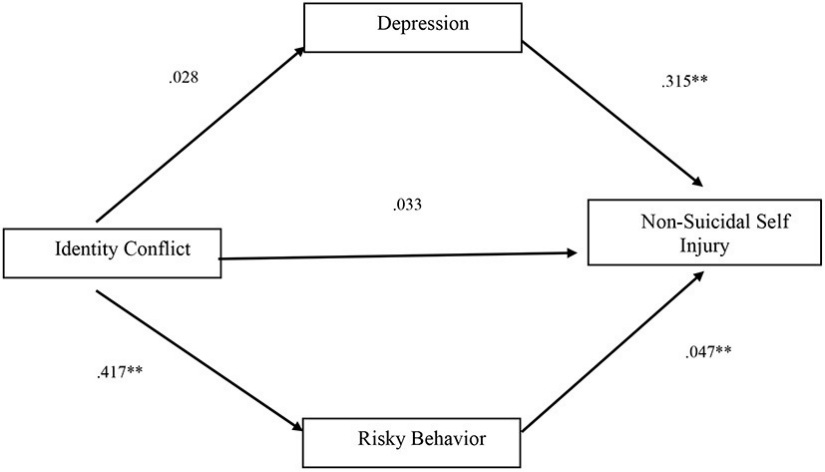
Pathways to non-suicide self-injury among druze adolescents. **p < 0.01. *Note*. Standardized regression coefficients are presented.

A mediation analysis was conducted *via* SPSS Process (42) to examine the mechanisms underlying the association between identity conflict and NSSI. The full model was significant, F_(3, 200)_ = 38.94, *p* < 0.001, r^2^ = 0.37 (Figure 2), revealing that identity conflict has a significant indirect effect on NSSI through risky behavior, β = 0.019, *p* < 0.05 CI [0.007, 0.038] and a significant total effect on NSSI β = 0.061, *p* < 0.05 CI [0.012, 0.010]. The direct effect on NSSI (β = 0.033, *p* = *0.115* CI [−0.008, 0.073]) and indirect effect through depression (β = 0.009 *p* = *0.4*5 CI [−0.017, 0.040]) were not significant.

## Discussion

In this study, we examined the prevalence of and risk factors for NSSI among Druze adolescents in Israel, and explored, among this group, the effect of identity conflict on engagement in NSSI. In total 19.7% of the sample reported having engaged in NSSI, with boys being more prone to do so than girls. The results also indicated that adolescents who engage in NSSI, when compared to those who do not engage in NSSI, more frequently experience difficulties regarding sleep, have stronger symptoms of depression and anxiety, and show greater involvement in risky behaviors.

The most robust finding in our study is that Druze adolescents who engage in NSSI have greater identity conflict when compared to those who do not. A possible explanation for this may relate to what Harris (43) refers to as a “protective factor” in immigrant families. When compared to third-generation immigrants, first- and second-generation immigrants are more likely to live in families that maintain their traditional culture; these traditions and family connections serve as a buffer and “protect” immigrant youths from engaging in risky behaviors (44) and adopting “bad” indigenous behaviors (43, 45). Similarly, developing an integrated ethnic identity may reduce the existence of identity conflict in minorities. This is supported by findings indicating that positive ethnic identity predicts greater wellbeing for members of ethnic minorities (46).

While a link between identity conflict and engagement in risky behaviors has been found in multiple studies, our study findings indicate the existence of a lesser-known mediation effect, with identity conflict influencing NSSI through risky behavior. Based on previous research indicating that increased impulsivity is related to increased engagement in a variety of risky behaviors (29, 30), we postulated that impulsivity may be a mechanism underlying the link between engagement in risky behaviors and NSSI. A previous meta-analysis also demonstrated that individuals who engage in NSSI report greater impulsivity than individuals who do not (39). Moreover, similar to NSSI, impulsive behaviors are likely to be reinforced over time if they provide relief from negative emotions (47).

In this study, engagement in risky behaviors was found to be a mediator between identity conflict and NSSI; this accords with existing results. For example, one previous study found that adolescents with identity confusion (i.e., reconsideration of identity commitment/diffusion) report higher levels of delinquency (substance and alcohol abuse, etc.) than counterparts with more consolidated identities (48). Furthermore, identity confusion (diffusion) has been found to be related to several types of risky behaviors, such as drug and alcohol abuse (49). Meanwhile, there is evidence that engagement in risky behaviors can have a negative effect on identity formation. A longitudinal study found that adolescents with a high risk of externalizing problems have difficulty forming their identities in later years (50).

Interestingly, our findings indicate that depression was not associated with identity conflict and did not mediate the association between identity conflict and NSSI. This finding might emphasize the distinctive nature of risk factors related to self-injury among ethnic minorities. Research has previously indicated that while depression is a strong indicator of self-injury among Western cultures, aggression, impulsivity and health risk behaviors might be a stronger indicator of self-injury among disadvantaged ethnic minorities, especially among adolescents facing minority-related stressors such as identity conflict (21). Our findings emphasize the importance of focusing on externalized expressions of distress, rather than internalized, when considering culturally competent risk assessment (21, 51).

Our results regarding emotion-regulation are complex. As noted above, emotion-regulation is comprised of two distinct components: reappraisal and suppression. Expressive suppression was found to be correlated with NSSI when controlling for gender. This might indicate and reflect the known differences between boys and girls in suppression (52–54) that in turn, might influence the overall association between suppression and NSSI. An inverse correlation was found between cognitive emotion-regulation (reappraisal) and NSSI. These results are consistent with those of other studies (27), and indicate that increased usage of cognitive emotion-regulation is linked to a decreased probability of engaging in NSSI. This may imply that engagement in NSSI and the use of cognitive reappraisal are alternative techniques for reducing stress. Consequently, teaching cognitive reappraisal techniques to adolescents may reduce their risk of engaging in NSSI; however, further research is needed to validate this connection.

Additionally, NSSI plays a role not only in reducing the negative emotional distress associated with identity conflict, but also in altering negative self-appraisal. NSSI is linked to low self-esteem (55), and this may explain why those who engage in NSSI focus on their negative emotions. More specifically, for individuals who engage in NSSI, NSSI is linked to their self-perception of being deeply flawed and deficient (14). Thus, causing injury to the body may represent a means of managing negative self-appraisal and associated painful emotions. Further, NSSI has been found to be a defense mechanism against “loss of self” and dissociation (14). Thus, for individuals with a dissociative identity disorder engaging in NSSI can be a means of forging links between multiple “selves” (14). Such linkage can manifest physically in the form of a scar that is visible throughout changes in the self over time. In conclusion, engagement in self-harm may help individuals who are experiencing complicated and confusing situations, such as identity conflict, define their identities.

## Limitations

This study has several limitations. Although well-validated measures of assessing NSSI and other correlates were used, the results rely on the assumption that the adolescents were forthcoming regarding their behavioral, psychological, and emotional difficulties. Secondly, the cross-sectional design used meant we could not analyze causation or the temporal effect of identity on depression and NSSI. Accordingly, future longitudinal designs should examine these overlapping relationships.

## Conclusions and implications

To the best of our knowledge, this study is the first to focus on adolescent Druze as an at-risk population for engaging in NSSI. We specifically examined the role of identity conflict and other established risk factors for NSSI (depression, risky behaviors, emotion regulation, and sleep problems) in engagement in NSSI among this population. Given that previous research has found a correlation between NSSI and suicide attempts (56), identifying populations that are prone to engaging in NSSI has significant value in regard to reducing suicide risk and distress. Our findings revealed a strong connection between identity conflict and NSSI, and showed that this relationship is mediated through engagement in risky behaviors. From a clinical perspective, it is possible that consolidation of adolescents' identity can help reduce both risky behaviors, depression and engagement in NSSI. Our findings indicate that developing strong ethnic identity in minorities has a buffering effect on the relationship between psychosocial risk factors and engagement in risky behaviors (57, 58). Thus, we assume that the development of a strong ethnic identity in adolescents from minorities may serve as a buffer on the relationship between identity conflict, engagement in risky behaviors, and depression and, consequently, may also help reduce engagement in NSSI. Further research is needed to determine whether it is possible to reduce symptoms of depression and engagement in risky behaviors by means of therapy that focuses on formatting identity in a way that reduces identity conflict.

## Data availability statement

The raw data supporting the conclusions of this article will be made available by the authors, without undue reservation.

## Ethics statement

The studies involving human participants were reviewed and approved by Ethics Committee of the Ministry of Education of Israel and the institutional review board of the Academic College of Tel-Aviv-Yaffo. Written informed consent to participate in this study was provided by the participants' legal guardian/next of kin.

## Author contributions

NT: writing—original draft: preparation, creation and/or presentation of the published work, specifically writing the initial draft (including substantive translation) and writing—review & editing: preparation, creation and/or presentation of the published work by those from the original research group, specifically critical review, and commentary or revision—including pre- or post-publication stages. SO: writing—original draft: preparation, creation and/or presentation of the published work and specifically writing the initial draft (including substantive translation). YS: formal analysis: application of statistical, mathematical, computational, or other formal techniques to analyze or synthesize study data. SHal: conceptualization: ideas, formulation or evolution of overarching research goals and aims, funding acquisition: acquisition of the financial support for the project leading to this publication, and supervision: oversight and leadership responsibility for the research activity planning and execution and including mentorship external to the core team. SHam: conceptualization: ideas, formulation or evolution of overarching research goals and aims, investigation: conducting a research and investigation process, specifically performing the experiments, or data/evidence collection and management, and coordination responsibility for the research activity planning and execution, resources: provision of study materials, reagents, materials, patients, laboratory samples, animals, instrumentation, computing resources, or other analysis tools, and supervision: oversight and leadership responsibility for the research activity planning and execution and including mentorship external to the core team. NT and SO: equally contributed to the preparation and writing of the paper, as agreed by all other authors. All authors contributed to the article and approved the submitted version.

## Conflict of interest

The authors declare that the research was conducted in the absence of any commercial or financial relationships that could be construed as a potential conflict of interest.

## Publisher's note

All claims expressed in this article are solely those of the authors and do not necessarily represent those of their affiliated organizations, or those of the publisher, the editors and the reviewers. Any product that may be evaluated in this article, or claim that may be made by its manufacturer, is not guaranteed or endorsed by the publisher.
